# Supplementary material for: Double gravity-assist rendezvous trajectory to Halley’s comet using deep-space low thrust
Source: PLoS One. 2026 Nov 4;21(11):e0361882. doi: 10.1371/journal.pone.0361882 (PMC13734607; doi:10.1371/journal.pone.0361882)
Supplement: Peer review history

# Peer review: Decision Letter 0

**DOI:** 10.1371/journal.pone.0361882.r001

**Authors:** Guangyu Sun

13 Sep 2026

Dear Dr. Fantino,

Thank you for submitting your manuscript to PLOS One. After careful consideration, we feel that it has merit but does not fully meet PLOS One’s publication criteria as it currently stands. Therefore, we invite you to submit a revised version of the manuscript that addresses the points raised during the review process.

Please submit your revised manuscript by Oct 13 2026 11:59PM. If you will need more time than this to complete your revisions, please reply to this message or contact the journal office at plosone@plos.org. When you're ready to submit your revision, log on to https://www.editorialmanager.com/pone/ and select the 'Submissions Needing Revision' folder to locate your manuscript file.

- A letter that responds to each point raised by the academic editor and reviewer(s). You should upload this letter as a separate file labeled 'Response to Reviewers'.
- A marked-up copy of your manuscript that highlights changes made to the original version. You should upload this as a separate file labeled 'Revised Manuscript with Track Changes'.
- An unmarked version of your revised paper without tracked changes. You should upload this as a separate file labeled 'Manuscript'.

If you would like to make changes to your financial disclosure, please include your updated statement in your cover letter. Guidelines for resubmitting your figure files are available below the reviewer comments at the end of this letter.

If applicable, we recommend that you deposit your laboratory protocols in protocols.io to enhance the reproducibility of your results. Protocols.io assigns your protocol its own identifier (DOI) so that it can be cited independently in the future. For instructions see: https://journals.plos.org/plosone/s/submission-guidelines#loc-laboratory-protocols. Additionally, PLOS One offers an option for publishing peer-reviewed Lab Protocol articles, which describe protocols hosted on protocols.io. Read more information on sharing protocols at https://plos.org/protocols?utm_medium=editorial-email&utm_source=authorletters&utm_campaign=protocols.

As the corresponding author, your ORCID iD is verified in the submission system and will appear in the published article. PLOS supports the use of ORCID, and we encourage all coauthors to register for an ORCID iD and use it as well. Please encourage your coauthors to verify their ORCID iD within the submission system before final acceptance, as unverified ORCID iDs will not appear in the published article. *Only*  the individual author can complete the verification step; PLOS staff *cannot*  verify ORCID iDs on behalf of authors.

We look forward to receiving your revised manuscript.

Kind regards,

Guangyu Sun, Ph.D

Academic Editor

PLOS One

**Journal Requirements:**

When submitting your revision, we need you to address these additional requirements.

1. Please ensure that your manuscript meets PLOS ONE's style requirements, including those for file naming. The PLOS ONE style templates can be found at

https://journals.plos.org/plosone/s/file?id=wjVg/PLOSOne_formatting_sample_main_body.pdf and

https://journals.plos.org/plosone/s/file?id=ba62/PLOSOne_formatting_sample_title_authors_affiliations.pdf

2. We note that the grant information you provided in the ‘Funding Information’ and ‘Financial Disclosure’ sections do not match.

When you resubmit, please ensure that you provide the correct grant numbers for the awards you received for your study in the ‘Funding Information’ section.

3. Thank you for stating the following financial disclosure:

“R. Flores and E. Fantino received support from the Polar Research Center (PRC) of Khalifa University of Science and Technology (KU) and grant ELLIPSE/8434000533 from Abu Dhabi's Technology Innovation Institute (TII).

A. Beolchi, C. Pozzi and C. Barbieri acknowledge KU's internal grant CIRA-2021-65/8474000413.

URLs:

KU - https://ku.ac.ae/

TII - https://www.tii.ae/”

Please state what role the funders took in the study.  If the funders had no role, please state: "The funders had no role in study design, data collection and analysis, decision to publish, or preparation of the manuscript."

If this statement is not correct you must amend it as needed.

Please include this amended Role of Funder statement in your cover letter; we will change the online submission form on your behalf.

4. If the reviewer comments include a recommendation to cite specific previously published works, please review and evaluate these publications to determine whether they are relevant and should be cited. There is no requirement to cite these works unless the editor has indicated otherwise.

5. Please review your reference list to ensure that it is complete and correct. If you have cited papers that have been retracted, please include the rationale for doing so in the manuscript text, or remove these references and replace them with relevant current references. Any changes to the reference list should be mentioned in the rebuttal letter that accompanies your revised manuscript. If you need to cite a retracted article, indicate the article’s retracted status in the References list and also include a citation and full reference for the retraction notice.

[Note: HTML markup is below. Please do not edit.]

Reviewers' comments:

Reviewer's Responses to Questions

**Comments to the Author**

1. Is the manuscript technically sound, and do the data support the conclusions?

Reviewer #1: Yes

Reviewer #2: Yes

**********

2. Has the statistical analysis been performed appropriately and rigorously?

Reviewer #1: Yes

Reviewer #2: Yes

**********

3. Have the authors made all data underlying the findings in their manuscript fully available?

The PLOS Data policy

Reviewer #1: Yes

Reviewer #2: Yes

**********

4. Is the manuscript presented in an intelligible fashion and written in standard English?

Reviewer #1: Yes

Reviewer #2: Yes

**********

Reviewer #1: To Authors

This manuscript presents a trajectory design for comet Halley rendezvous using deep-space low-thrust propulsion and double gravity assists. The topic is relevant and the methodology is generally sound. However, several issues regarding assumptions, reproducibility, and clarity need to be addressed before publication.

Reviewer #2: Good, timely work. The three-parameter reduction and the closed-form flyby geometry are elegant, and the 2030–2040 sweep is a useful result. A few things to fix.

1. Power and thruster budget. The 640 W input is assumed, not budgeted. Please add a short table showing how many RTG units are needed to hold that power through the Saturn–Halley leg after ~24 years of degradation, and the total impulse demanded of the Hall thruster against demonstrated throughput. If the numbers close, the "proven technology" claim is much stronger for having been shown. If they don't, I'd soften the wording to something like "avoids reliance on high-power nuclear-electric propulsion", which your results already support.

2. Numbers in Section 3.2.1. The impulsive total for 2036 is 7.89 km/s (Table 3); 8.81 belongs to the 2037 case. The 75 kg chemical-propulsion figure follows from the wrong value and should be recomputed. Conclusion unaffected.

3. Eq. 2. The thrust term needs to be divided by the spacecraft mass.

4. Saturn periapsis. 80,000 km sits radially inside the C ring, and since the flyby angles come from ΔV minimisation alone, the ring-plane crossing radius is an uncontrolled outcome. Please report it for both solutions.

5. Jupiter flyby. The 2036 case passes at 2.08 R_J, deep in the belts, with 22 years of operation still ahead. A total dose estimate would help. The 2037 solution at 6.5 R_J is much easier on the spacecraft, which may be worth a sentence.

6. Reproducibility. Add a table of full Cartesian states (TDB epoch, r, v) at events 1–6 so the trajectories can be checked independently.

**********

what does this mean?). If published, this will include your full peer review and any attached files.

If you choose “no”, your identity will remain anonymous but your review may still be made public.

**Do you want your identity to be public for this peer review?**  For information about this choice, including consent withdrawal, please see our Privacy Policy

Reviewer #1: No

Reviewer #2: **Yes:**  Raul Bonadia Rodrigues

**********

[NOTE: If reviewer comments were submitted as an attachment file, they will be attached to this email and accessible via the submission site. Please log into your account, locate the manuscript record, and check for the action link "View Attachments". If this link does not appear, there are no attachment files.]

To ensure your figures meet our technical requirements, please review our figure guidelines: https://journals.plos.org/plosone/s/figures

You may also use PLOS’s free figure tool, NAAS, to help you prepare publication quality figures: https://journals.plos.org/plosone/s/figures#loc-tools-for-figure-preparation.

NAAS will assess whether your figures meet our technical requirements by comparing each figure against our figure specifications.

# Author comment: Author response to Decision Letter 1

**DOI:** 10.1371/journal.pone.0361882.r002

5 Oct 2026

The authors wish to thank the editorial team and the referees for their time and effort going through the manuscript, and their suggestions for improvement. Below you will find the replies to all the comments from the reviewers. Changes to the manuscript are highlighted in purple to make them apparent. Note that properly addressing the comments led us to change the propulsion system model. This, in turn, required recalculating all data. Therefore, even though they look similar, Figures 4 to 17 and Tables 4 to 7 are new. The Supporting Information file is also completely different.

Reviewer #1: To Authors

C1.1 - This manuscript presents a trajectory design for comet Halley rendezvous using deep-space low-thrust propulsion and double gravity assists. The topic is relevant and the methodology is generally sound. However, several issues regarding assumptions, reproducibility, and clarity need to be addressed before publication.

A1.1 - Given that no specific problems are mentioned, we understand that the issues are those signaled by Reviewer #2. Please see the answers below.

Reviewer #2: Good, timely work. The three-parameter reduction and the closed-form flyby geometry are elegant, and the 2030–2040 sweep is a useful result. A few things to fix.

C2.1 - Power and thruster budget. The 640 W input is assumed, not budgeted. Please add a short table showing how many RTG units are needed to hold that power through the Saturn–Halley leg after ~24 years of degradation, and the total impulse demanded of the Hall thruster against demonstrated throughput. If the numbers close, the "proven technology" claim is much stronger for having been shown. If they don't, I'd soften the wording to something like "avoids reliance on high-power nuclear-electric propulsion", which your results already support.

A2.1 - To ensure that the calculations correspond to truly "proven technology" we have modeled the power source as three GPHS-RTGs, including the decay of the heat source and reserving a budget of 100 W for critical systems that have to remain active while the motor operates. To ensure that the throughput of the thruster is adequate, we selected NEXT, which has demonstrated a capacity over 900 kg (this is just the value at the end of the test campaign, the actual throughput is likely much higher). Furthermore, it is flight-proven, having powered DART. NEXT is not the best option from the performance standpoint, because it has to operate at the bottom end of its throttling range. However, more convenient alternatives like HETs have not yet proven the necessary throughput. The variation of thrust and specific impulse as the RTGs decay has been taken into account. See section 2.2 for details.

Obviously, changing the propulsion system model required recalculating everything. All the data in the paper are new, so large sections of the manuscript have been marked accordingly. However, apart from matters related to the propulsion plant model, which is completely different, the changes are mainly numerical values and adaptation of the discussion to the new data. The substance of the paper does not change because, as the reviewer shall see, the variations in propellant budget and event dates are quite limited. Most of the differences can be traced back to degraded performance in the last leg, due to RTG decay.

C2.2 - Numbers in Section 3.2.1. The impulsive total for 2036 is 7.89 km/s (Table 3); 8.81 belongs to the 2037 case. The 75 kg chemical-propulsion figure follows from the wrong value and should be recomputed. Conclusion unaffected.

A2.2 - Thank you for spotting the typo, it was indeed the wrong number. We double-checked the new version which, due to the new data, corresponds to a slightly different value.

C2.3 - Eq. 2. The thrust term needs to be divided by the spacecraft mass.

A2.3 - Again, thank you for detecting the mistake, we fixed the typo.

C2.4 - Saturn periapsis. 80,000 km sits radially inside the C ring, and since the flyby angles come from ΔV minimisation alone, the ring-plane crossing radius is an uncontrolled outcome. Please report it for both solutions.

A2.4 - We have included the ring crossings in the discussion. The trajectory intersects the rings much farther from the planet than the periapsis, the radius is between 150,000 and 310,000 kmn depending on the branch of the hyperbola (inbound/outbound) and launch date. See lines 446-448 and 473-474 of the manuscript.

C2.5 - Jupiter flyby. The 2036 case passes at 2.08 R_J, deep in the belts, with 22 years of operation still ahead. A total dose estimate would help. The 2037 solution at 6.5 R_J is much easier on the spacecraft, which may be worth a sentence.

A2.5 - Estimating the effects of radiation would require, at the very least, a preliminary design of the spacecraft. Unfortunately, that is not yet available because this is simply a trajectory analysis to validate the dynamical feasibility of the EJSH transfer. We added a comment indicating that it is a topic that deserves careful consideration should the concept be ever developed, but we believe that it falls outside the scope of the manuscript. We have also indicated that the viability of such a flyby was demostrated long ago by Pioneer 11 (in fact, in went even deeper into the radiation belts), so it should not be a major impediment. Furthermore, the duration of the flyby is short, which limits the exposure, and the majority of electronic systems can be switched off for protection, since the maneuver is unpowered. See lines 453-462 and 475.

C2.6 - Reproducibility. Add a table of full Cartesian states (TDB epoch, r, v) at events 1–6 so the trajectories can be checked independently.

A2.6 - A table (in machine-readable format, which is more convenient for thrid-party validation) is included for each trajectory in the Supporting Information file. It contains time (Julian date), position and velocity in Cartesian coordinates, mass and thrust vector. The table contains not only the events, but also 99 intermediate points of each leg (because the LT transcription uses 100 steps per leg). We added a comment in the text to highlight this fact (lines 438-439). Also, we included additional information in tables 6 & 7 (e.g., flyby angles and hyperbolic excess speed) to improve reproducibility.

We thank the reviewer for his valuable comments and suggestions that, while laborious to implement, resulted in a much more solid manuscript.

Attachment Submitted filename: Reply_to_reviewers_V2.txt

# Peer review: Decision Letter 1

**DOI:** 10.1371/journal.pone.0361882.r003

**Authors:** Guangyu Sun

19 Oct 2026

Double gravity-assist rendezvous trajectory to Halley's comet using deep-space low thrust

PONE-D-26-41206R1

Dear Dr. Fantino,

We’re pleased to inform you that your manuscript has been judged scientifically suitable for publication and will be formally accepted for publication once it meets all outstanding technical requirements.

Within one week, you’ll receive an e-mail detailing the required amendments. When these have been addressed, you’ll receive a formal acceptance letter and your manuscript will be scheduled for publication.

An invoice will be generated when your article is formally accepted. Please note, if your institution has a publishing partnership with PLOS and your article meets the relevant criteria, all or part of your publication costs will be covered. Please make sure your user information is up-to-date by logging into Editorial Manager at Editorial Manager® and clicking the ‘Update My Information' link at the top of the page. For questions related to billing, please contact billing support.

If your institution or institutions have a press office, please notify them about your upcoming paper to help maximize its impact. If they’ll be preparing press materials, please inform our press team as soon as possible -- no later than 48 hours after receiving the formal acceptance. Your manuscript will remain under strict press embargo until 2 pm Eastern Time on the date of publication. For more information, please contact onepress@plos.org.

Kind regards,

Guangyu Sun, Ph.D

Academic Editor

PLOS One

Additional Editor Comments (optional):

Reviewers' comments:

Reviewer's Responses to Questions

**Comments to the Author**

Reviewer #2: All comments have been addressed

**********

2. Is the manuscript technically sound, and do the data support the conclusions?

Reviewer #2: Yes

**********

3. Has the statistical analysis been performed appropriately and rigorously?

Reviewer #2: Yes

**********

4. Have the authors made all data underlying the findings in their manuscript fully available?

The PLOS Data policy

Reviewer #2: Yes

**********

5. Is the manuscript presented in an intelligible fashion and written in standard English?

Reviewer #2: Yes

**********

Reviewer #2: (No Response)

**********

what does this mean?). If published, this will include your full peer review and any attached files.

If you choose “no”, your identity will remain anonymous but your review may still be made public.

**Do you want your identity to be public for this peer review?**  For information about this choice, including consent withdrawal, please see our Privacy Policy

Reviewer #2: **Yes:**  Raul Bonadia Rodrigues

**********

# Editor report: Acceptance letter

**DOI:** 10.1371/journal.pone.0361882.r004

**Authors:** Guangyu Sun

PONE-D-26-41206R1

PLOS One

Dear Dr. Fantino,

I'm pleased to inform you that your manuscript has been deemed suitable for publication in PLOS One. Congratulations! Your manuscript is now being handed over to our production team.

At this stage, our production department will prepare your paper for publication. This includes ensuring the following:

* All references, tables, and figures are properly cited

* All relevant supporting information is included in the manuscript submission,

* There are no issues that prevent the paper from being properly typeset

You will receive further instructions from the production team, including instructions on how to review your proof when it is ready. Please keep in mind that we are working through a large volume of accepted articles, so please give us a few days to review your paper and let you know the next and final steps.

Lastly, if your institution or institutions have a press office, please let them know about your upcoming paper now to help maximize its impact. If they'll be preparing press materials, please inform our press team within the next 48 hours. Your manuscript will remain under strict press embargo until 2 pm Eastern Time on the date of publication. For more information, please contact onepress@plos.org.

You will receive an invoice from PLOS for your publication fee after your manuscript has reached the completed accept phase. If you receive an email requesting payment before acceptance or for any other service, this may be a phishing scheme. Learn how to identify phishing emails and protect your accounts at https://explore.plos.org/phishing.

If we can help with anything else, please email us at customercare@plos.org.

Thank you for submitting your work to PLOS One and supporting open access.

Kind regards,

PLOS One Editorial Office Staff

on behalf of

Dr. Guangyu Sun

Academic Editor

PLOS One
